# Cyclic GMP protects human macrophages against peroxynitrite-induced apoptosis

**DOI:** 10.1186/1476-9255-6-14

**Published:** 2009-05-07

**Authors:** Catherine A Shaw, David J Webb, Adriano G Rossi, Ian L Megson

**Affiliations:** 1 Centre for Cardiovascular Science, The Queen's Medical Research Institute, University of Edinburgh, Edinburgh, UK; 2 MRC Centre for Inflammation Research, The Queen's Medical Research Institute, University of Edinburgh, Edinburgh, UK; 3 Free Radical Research Facility, Department of Diabetes and Cardiovascular Science, UHI Millennium Institute, Inverness, UK

## Abstract

**Background:**

Nitric oxide (NO) can be both pro- and anti-apoptotic in various cell types, including macrophages. This apparent paradox may result from the actions of NO-related species generated in the microenvironment of the cell, for example the formation of peroxynitrite (ONOO^-^). In this study we have examined the ability of NO and ONOO^- ^to evoke apoptosis in human monocyte-derived macrophages (MDMϕ), and investigated whether preconditioning by cyclic guanosine monophosphate (cGMP) is able to limit apoptosis in this cell type.

**Methods:**

Characterisation of the NO-related species generated by (Z)-1- [2-(2-aminoethyl)-N-(2-ammonioethyl)amino]diazen-1-ium-1,2-diolate (DETA/NO) and 1,2,3,4-oxatriazolium, 5-amino-3-(3,4-dichlorophenyl)-, chloride (GEA-3162) was performed by electrochemistry using an isolated NO electrode and electron paramagnetic resonance (EPR) spectrometry. Mononuclear cells were isolated from peripheral blood of healthy volunteers and cultured to allow differentiation into MDMϕ. Resultant MDMϕ were treated for 24 h with DETA/NO (100 – 1000 μM) or GEA-3162 (10 – 300 μM) in the presence or absence of BAY 41–2272 (1 μM), isobutylmethylxanthine (IBMX; 1 μM), 1H- [1,2,4]oxadiazolo [4,3-a]quinoxalin-1-one (ODQ; 20 μM) or 8-bromo-cGMP (1 mM). Apoptosis in MDMϕ was assessed by flow cytometric analysis of annexin V binding in combination with propidium iodide staining.

**Results:**

Electrochemistry and EPR revealed that DETA/NO liberated free NO radical, whilst GEA-3162 concomitantly released NO and O_2_^-^, and is therefore a ONOO^- ^generator. NO (DETA/NO) had no effect on cell viability, but ONOO^- ^(GEA-3162) caused a concentration-dependent induction of apoptosis in MDMϕ. Preconditioning of MDMϕ with NO in combination with the phosphodiesterase inhibitor, 3-Isobutyl-1-methylxanthine (IBMX), or the NO-independent stimulator of soluble guanylate cyclase, BAY 41–2272, significantly attenuated ONOO^-^-induced apoptosis in a cGMP-dependent manner.

**Conclusion:**

These results demonstrate disparities between the ability of NO and ONOO^- ^to induce apoptosis in human MDMϕ. Furthermore, this study provides evidence for a novel cGMP-dependent pre-conditioning mechanism to limit ONOO^-^-induced apoptosis in human MDMϕ.

## Background

Apoptosis is a highly regulated and fundamental biological process governing cell survival. During apoptosis, the integrity of the cell membrane is maintained, therefore preventing release of the histotoxic cell contents. Because apoptotic cells are instantly recognised by phagocytes and removed from the inflammatory site, successful apoptosis is now recognised to be crucial to the resolution of inflammation. Failure of inflammatory cells to undergo apoptosis, or failure of subsequent phagocytic removal of apoptotic cells is believed to result in incomplete resolution and an exacerbation of the inflammatory response [1-4]. Thus, apoptosis is a non-inflammatory mechanism for the removal of inflammatory cells from a site of tissue damage.

The propensity of a cell to undergo apoptosis is determined by the net balance of many pro- and anti-apoptotic exogenous and endogenous factors [5-7]. The signalling molecule, nitric oxide (NO), has previously been reported to induce apoptosis in various cell types, including macrophages [8-12]. However, the role of NO in apoptosis is complicated by a number of reports indicating that it can be both pro- and anti-apoptotic [13-15]. The generally accepted paradigm is that lower NO concentrations produced constitutively by endothelial NO synthase (eNOS) and neuronal NO synthase (nNOS) are cytoprotective via primarily cGMP-dependent mechanisms, whilst higher, supraphysiological concentrations generated in some pathologies by the inducible form of NOS (iNOS) mediate apoptosis via mechanisms independent of cGMP signalling [16].

This apparent paradox may be explained, at least in part, by the production of intermediary NO-related species, with the ultimate outcome of any NO-mediated response being dependent on the precise NO-related species formed in the microenvironment, as well as the cell type in question. In biological systems NO often reacts with superoxide anions (O_2_^•-^), resulting in the formation of the powerful oxidising agent, peroxynitrite (ONOO^-^) [17-20]. ONOO^- ^has been shown to induce apoptosis in human inflammatory cells, such as neutrophils [21,22].

Disparities between the sensitivity of different cell types to apoptosis induced by NO (or NO-related species), suggests the presence of protective mechanisms in those cell types resistant to NO-evoked apoptosis. Such protective mechanisms may depend on the anti-apoptotic qualities of NO itself. Indeed, non-toxic concentrations of NO, and agents that act to elevate cGMP independently of NO, have been demonstrated to protect rodent macrophages and vascular smooth muscle cells (VSMC) against subsequent NO-induced cell death [23-26].

Here, we test the hypothesis that the NO-related species, ONOO^-^, but not NO, induces apoptosis in human macrophages derived from the monocyte population of peripheral blood. Furthermore, we investigate whether low concentrations of NO, and the novel NO-independent stimulator of soluble guanylate cyclase, BAY 41–2272, are able to precondition human macrophages against subsequent ONOO^-^-induced apoptosis.

## Methods

### Electrochemical Detection of NO

NO radical released in Iscove's modified Dulbecco's tissue culture medium (IMDM; Gibco Life Technologies, UK) from (Z)-1- [2-(2-aminoethyl)-N-(2-ammonioethyl)amino]diazen-1-ium-1,2-diolate (DETA/NO; Axxora Ltd, UK) and 1,2,3,4-oxatriazolium, 5-amino-3-(3,4-dichlorophenyl)-, chloride (GEA-3162; Axxora Ltd, UK) was measured by an isolated NO electrode (Iso-NO II, World Precision Instruments, UK). NO production by DETA/NO (300 μM) was recorded for 30 min.

Superoxide dismutase (SOD; 50–500 U.ml^-1^; Sigma-Aldrich, UK) was added cumulatively in stepwise increments to unmask NO produced by GEA-3162 (300 μM) [21]. Each bolus addition of SOD was added to the electrode chamber once the signal from the previous addition of SOD had reached a plateau, usually after approximately 3–5 min. Alternatively, GEA-3162 and 500 U.ml^-1 ^SOD were introduced simultaneously to the electrode chamber and the signal recorded until it decayed to baseline. Finally, because the structurally similar compound, SIN-1, can generate ONOO^- ^*in vitro*, but NO in the presence of biological tissues [27], we investigated whether GEA-3162 could generate NO in the presence of a suspension of monocyte-derived macrophages (1 × 10^6 ^cells/ml).

The NO scavenger, haemoglobin (Hb; 5 μM) [28], was introduced to the electrode chamber to confirm NO generation in experiments where the signal had not decayed to baseline at the end of the incubation period.

### Electron Paramagnetic Resonance Studies

The concentration of oxidising free radical species generated by GEA-3162 in the presence or absence of SOD was assessed in IMDM tissue culture medium by electron paramagnetic resonance (EPR) spectrometry (electron spin resonance). GEA-3162 (10–300 μM) was incubated for 30 min at 37°C in the presence or absence of SOD (500 U.ml^-1^) plus the chemical spin trap Tempone-H hydrochloride (1 mM; Axxora, UK; prepared in water containing EDTA (10 mM) [29,30]. The intensity of the EPR signals corresponding to the formation of the radical adduct, 4-oxo-tempo (triplet centred around 3360 G), were recorded using a Miniscope MS100 X-band spectrometer (Magnettech, Germany) with the following parameter settings: field sweep 51.2 G, microwave frequency 9.5 GHz, microwave power 20 mW, modulation amplitude 1500 mG. Control experiments, consisting of tempone-H in IMDM in the absence of radical-generating compounds, were found to generate small signals due to the auto-oxidation of tempone-H to 4-oxo-tempo; these control signals were subtracted from the corresponding experimental signals. There experiments were repeated with GEA-3162 which had undergone spontaneous decomposition at 37°C for 7 days.

### Cell Isolation and Culture

Mononuclear cells (MNC) and polymorphonuclear cells were isolated from human blood as previously described [31,32]. Briefly, whole, citrated blood was centrifuged (200 × *g*; 20 min) and platelet-rich plasma (PRP) aspirated. Leukocytes were separated from erythrocytes by dextran sedimentation, then further divided into MNC and granulocyte populations by centrifugation through a discontinuous Percoll^® ^(Pharamcia, UK) gradient (720 × *g*; 20 min). MNC were harvested at the 55:68% interface and re-suspended at a density of 4 × 10^6 ^cells/ml in IMDM supplemented with penicillin and streptomycin (both 100 U.ml^-1^), prior to enrichment for monocytes by selective adherence to 48-well (2 × 10^6 ^cells/well) tissue culture plates for 1 h at 37°C in 5% CO_2_. Adherent monocytes were washed three times in phosphate buffered saline (PBS) and then allowed to differentiate into MDMϕ for 5 days (37°C; 5% CO_2_) in IMDM containing penicillin/streptomycin and 10% autologous serum prepared by recalcification of  PRP. Medium was replaced every 2–3 days throughout the differentiation period.

### Experimental Protocols

In order to investigate the effect of NO radical and ONOO^- ^on cell viability, MDMϕ were treated for 24 h with the NO donor, DETA/NO (100–300 μM), the ONOO^- ^generator, GEA-3162 (10–300 μM), or vehicle control (DMSO; 1%) prior to assessment of apoptosis and necrosis by flow cytometry as described below. The experiments were repeated for GEA-3162 in the presence of the anti-oxidant enzymes SOD, or a combination of SOD plus catalase (both 500 U.ml^-1^). In order to investigate the actions of the breakdown products of the compound, additional experiments were conducted with GEA-3162 which had been allowed to decompose at 37°C for 7 days. The known pro-apoptotic agent, gliotoxin (1 μg.ml^-1^; Sigma Aldrich, UK) was used as a positive control for the assay [33-35].

To investigate the role of NO:cGMP signalling in ONOO^-^-induced apoptosis, MDMϕ were pre-treated for 24 h with DETA/NO (10 μM) plus either the NO-independent sGC stimulator, BAY 41–2272 (1 μM; Bayer, Germany; [36], or the non-specific phosphodiesterase (PDE) inhibitor, 3-isobutyl-1-methylxanthine (IBMX; 1 μM; Axxora, UK). BAY 41–2272 was used in combination with DETA/NO (10 μM) because recent evidence has suggested that, rather than acting via direct stimulation of sGC, the actions of this compound are a result of the synergistic effects of inhibition of PDE V coupled with sensitisation of sGC toward endogenous NO [37,38]. As IBMX also raises intracellular cGMP by inhibiting its breakdown, DETA/NO is used to provide the initial stimulus for cGMP production because human macrophages are unlikely to generate enough endogenous NO to result in cellular cGMP production. The ability of the NO:cGMP pathway to inhibit ONOO^- ^dependent apoptosis was further investigated by pre-treating MDMϕ for 24 h with the cell permeable cGMP analogue, 8-bromo-cGMP (1 mM), or a higher concentration of DETA/NO (300 μM) in the presence or absence of the sGC inhibitor, 1-*H*- [1,2,4]oxadiazolo [4,3-*a*]quinoxalin-1-one (ODQ; 20 μM).

Following this pre-treatment period, cells were washed twice in PBS to remove pre-treatment compounds prior to incubation for a further 24 h in the presence of the ONOO^- ^generator, GEA-3162 (100 μM). At this concentration, GEA-3162 induces significant apoptosis in MDMϕ without causing necrosis. Finally, a set of cells were treated for the final 24 h period only with the apoptotic agent, gliotoxin (1 μg.ml^-1^), which serves as a positive control for the assay.

### Flow Cytometric Analysis of Annexin V Binding and Propidium Iodide Staining

Cell death was assessed by flow cytometric detection of fluorescein isothiocyanate (FITC)-conjugated annexin V binding to phosphatiylserine (PS) exposed on the surface of apoptotic cells, or propidium iodide (PI) staining of late apoptotic/necrotic cells. Following the experimental protocol detailed above, cells were removed from tissue culture plates (0.25% trypsin with EDTA) and recovered cell suspensions incubated for 10 min on ice in the presence of FITC-annexin V in annexin V-binding buffer (prepared as a 1:500 dilution of annexin V in Hank's balanced salt solution (HBSS) containing 5 mM CaCl_2_). Following this incubation period, PI (final concentration 2 μg.ml^-1^) was added to the cell suspension/annexin V binding buffer mix for 1 min prior to analysis by Coulter EPICS XL flow cytometer (Beckman Coulter, USA) equipped with EXPO™ 32 data analysis software.

### Quantification of cGMP

Levels of cGMP produced by MDMϕ following incubation with DETA/NO in the absence and presence of BAY 41–2272 or IBMX were measured by enzyme-linked immunosorbant assay (ELISA; R&D Systems, UK) according to the manufacturer's instructions. Briefly, cell culture supernatants were aspirated and adherent cells lysed (2% Triton X 100). Recovered solutions were acidified (0.1 M HCl) and acetylated prior to performing the assay. Optical density (405 nm) was measured by a Multiskan Ascent plate reader and the concentration of cGMP was calculated from a standard curved produced from serial dilutions of acetylated cGMP solutions (0.08 – 50 pmol.ml^-1^). All standards and samples were assayed in duplicate.

### Statistics

Data are expressed throughout as mean ± SEM, with n values as indicated. Statistical tests are described in figure legends and were performed using GraphPad Prism software version 3.03 (GraphPad Software Inc., San Diego, USA). Results were considered to be statistically significant when *P *< 0.05.

## Results

### NO Release from DETA/NO and GEA-3162

**Figure 1:**
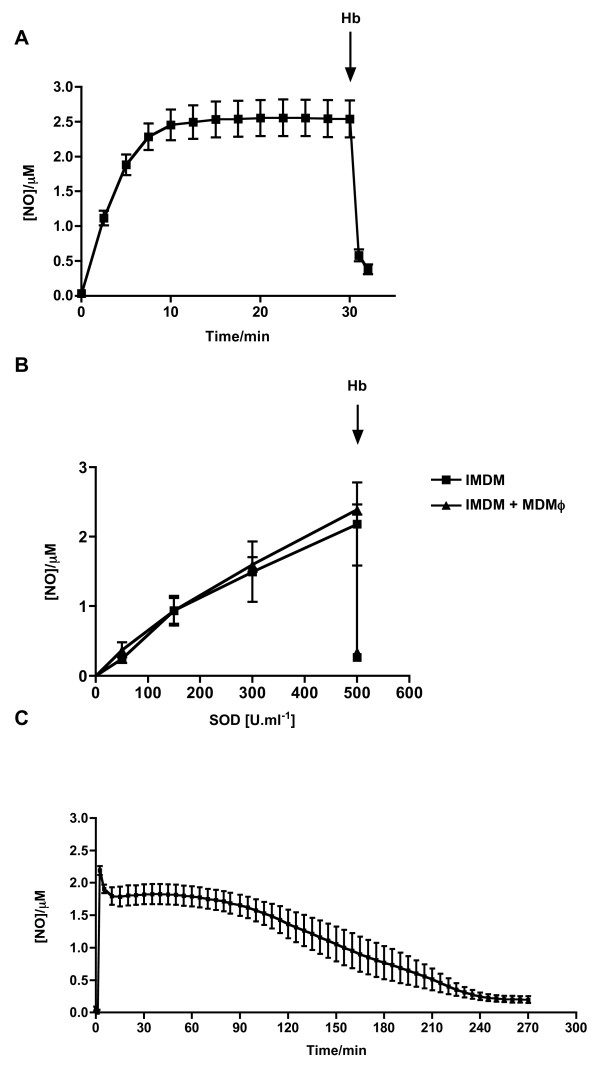
**NO generation by DETA/NO and GEA-3162 measured by isolated NO electrode** A – NO liberated by DETA/NO (300 μM) recorded for 30 min (n = 6). B – GEA-3162 (300 μM) failed to liberate free NO in solution except in the presence of SOD. Each addition of SOD is added approximately 3 – 5 min following the previous addition. The concentration of NO liberated in the presence of SOD was unchanged by MDMϕ (n = 3). Arrows indicate addition of Hb. C – In the presence of 500 U.ml^-1 ^SOD, GEA-3162 (300 μM) liberated NO over a period of approximately 4 h (n = 6).

DETA/NO (300 μM) generated a slow and prolonged NO release in IMDM that remained steady throughout a 30 min recording period. Introduction of the NO scavenger, haemoglobin (Hb; 5 μM), to the electrode chamber successfully quenched the signal from the electrode, confirming NO release from DETA/NO (figure 1A).

In common with previous findings [21], GEA-3162 (300 μM) failed to generate a detectable NO signal except in the presence of the superoxide scavenger, SOD (50 – 500 U.ml^-1^), when stepwise, incremental concentrations of NO were detectable. MDMϕ did not alter the inability of GEA-3162 to liberate NO in the absence of SOD, and did not alter concentration of NO released from GEA-3162 in the presence of SOD. Hb quenched the signal from GEA-3162 (figure 1B). In the presence of SOD to unmask the NO released by GEA-3162, NO decayed from a max value of 2.2 μM over a period of ~4 h (figure 1C).

### ONOO^- ^Generation by GEA-3162

**Figure 2:**
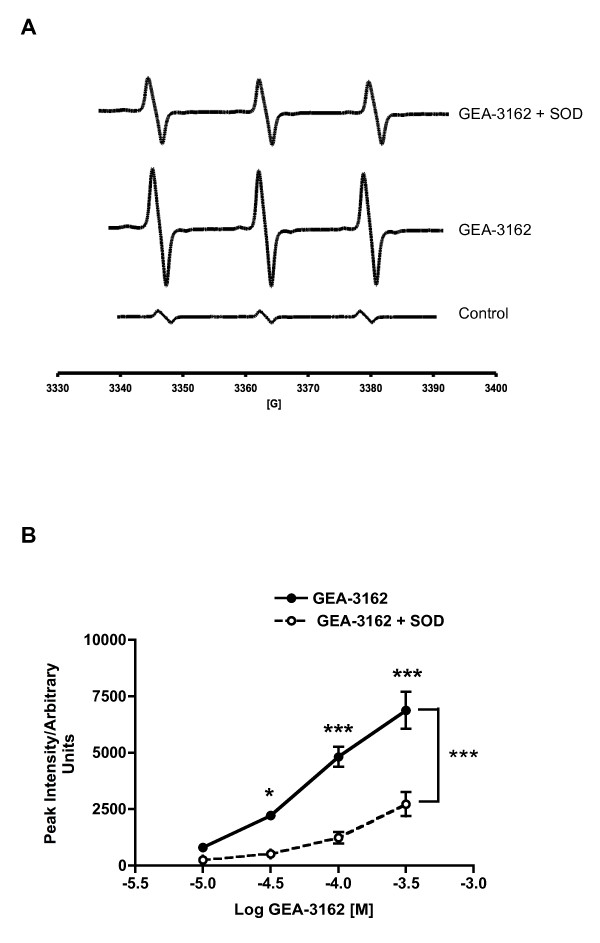
**Oxidising radical generation by GEA-3162**. A – representative EPR spectra generated by GEA-3162 (300 μM) in the presence and absence of SOD (500 U.ml^-1^). B – SOD reduced the intensity of the GEA-3162 EPR signal by a maximum of 61%. *P < 0.05, ***P < 0.001 (Two-way ANOVA followed by post hoc Bonferroni's test; n = 6).

GEA-3162 generated an EPR signal consistent with the production of the radical adduct, 4-oxo-tempo. SOD significantly attenuated the intensity of this signal by a maximum of 61% (figure 2). Incubation of GEA-3162 at 37°C for 7 days resulted in partial decomposition of the compound. The EPR signal from GEA-3162 treated in this way was diminished, but not completely abolished; the signal was reduced from an intensity of 6894 ± 810 (n = 6) to 2939 ± 650 (n = 3). This remaining signal was also quenched by SOD to 1443 ± 290.

### Quantification of cGMP

Levels of cGMP were undetectable (< 0.08 pmol.ml^-1^) in vehicle treated control MDMϕ. Those cells that had been treated with DETA/NO plus BAY 41–2272 or IBMX generated 0.325 and 0.40 pmol.ml^-1 ^cGMP per 1 × 10^6 ^cells respectively. Levels of cGMP generated by DETA/NO alone were below the limit of detection of the assay.

### Peroxynitrite, But Not NO, Induces Apoptosis in Human MDMϕ

**Figure 3:**
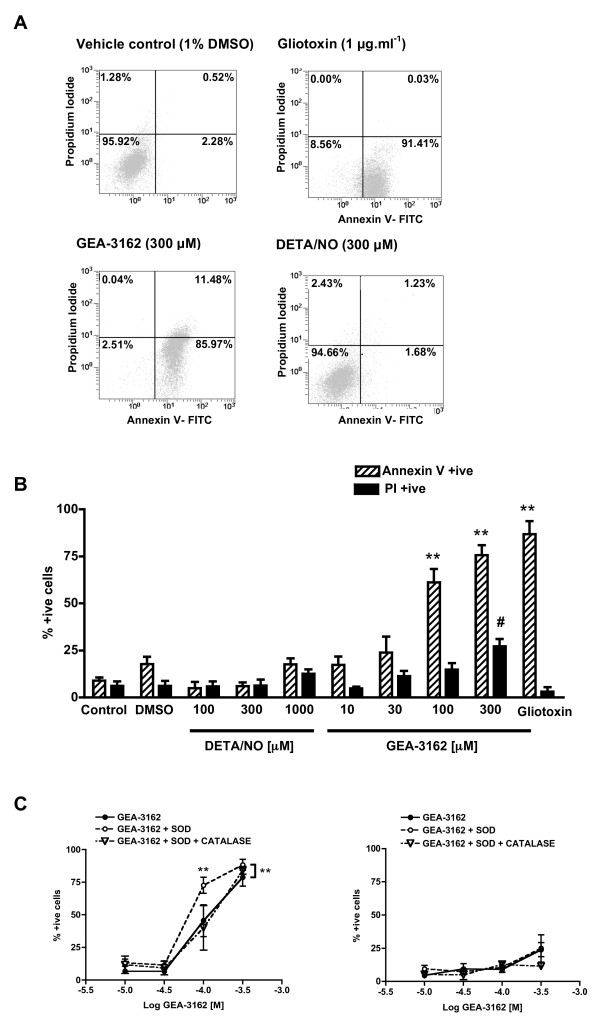
**GEA-3162 induces apoptosis in human MDMϕ**. A – representative flow cytometry plots. B – induction of apoptosis by GEA-3162 but not DETA/NO. **P < 0.01 significantly greater annexin V binding compared to control; #*P *< 0.05 significantly greater PI staining compared to control (unpaired, one-way ANOVA followed by post hoc Dunnett's test; n = 6 – 9). C – apoptosis (annexin V binding; left panel) induced by GEA-3162 was elevated by SOD (500 U.ml^-1^). A combination of SOD and catalase (both 500 U.ml^-1^) blunted the SOD-induced elevation of apoptosis but not reduce GEA-3162-induced apoptosis. PI staining (right panel) was unaffected by SOD or catalase. **P < 0.01; matched two ANOVA followed by post hoc Bonferroni's test; n = 6.

The NO donor, DETA/NO, at concentrations of up to 1 mM, had no affect on the levels of annexin V binding or PI staining in human MDMϕ. In contrast, the ONOO^- ^generator, GEA-3162 (10–300 μM) [21], caused a concentration-dependent induction of apoptosis in human MDMϕ, as demonstrated by an increase in annexin V binding (figure 3). At concentrations up to 100 μM, the increase in annexin V binding occurred in the absence of significant PI staining. However, at higher concentrations (300 μM) a significant level of PI staining was also observed. Therefore, in order to specifically induce apoptosis, a concentration of 100 μM GEA-3162 was selected for subsequent experiments. Whereas fresh GEA-3162 (100 μM) induced a maximum of 61 ± 7% (n = 9) apoptosis, this was reduced to 39 ± 9% (n = 3) following treatment with partially decomposed GEA-3162.

The level of apoptosis (annexin V binding) induced by GEA-3162 (10–300 μM) was further enhanced when MDMϕ were treated with GEA-3162 in the presence of SOD (figure 3C). The additional elevation of apoptosis induced by SOD was suppressed when MDMϕ were treated with GEA-3162 in the presence of a combination of SOD plus catalase (figure 3C). This combination did not, however, reduce overall levels of apoptosis induced by GEA-3162. Neither SOD nor catalase had any impact on the level of PI staining.

### cGMP Signalling Protects MDMϕ Against Peroxynitrite Induced Apoptosis

**Figure 4:**
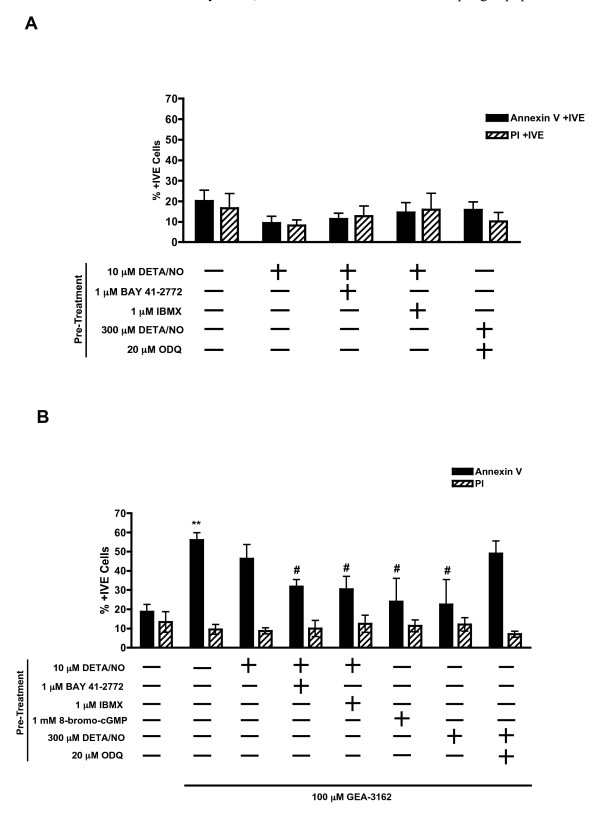
**cGMP signalling inhibits apoptosis**. MDMϕ were subjected to 24 h pre-treatment as shown below each column. A – The pre-treatments had no effect on cell viability after 24 h compared to vehicle (1% DMSO) treated cells (first column). B – The groups indicated were then treated for a further 24 h with GEA-3162 (100 μM). Pre-treatment with 8-bromo-cGMP, 300 μM DETA/NO, and 10 μM DETA/NO in combination with BAY 41–2272 or IBMX, all significantly attenuated GEA-3162-induced apoptosis. ODQ prevented the attenuation of apoptosis induced by 300 μM DETA/NO. **P < 0.01 significantly different from vehicle (1% DMSO) treated control cells (first column). #*P *< 0.01 significantly different from GEA-3162 treated cells (second column; one-way ANOVA followed by post hoc Dunnet's test; n = 3 – 9).

Control sets of cells demonstrated that 24 h pre-treatment of MDMϕ with DETA/NO (10 μM) in combination with BAY 41–2272 or IBMX (both 1 μM) had no effect on cell viability compared to untreated cells. Similarly, higher concentrations of DETA/NO (300 μM) alone or in combination with the sGC inhibitor, ODQ (20 μM), did not affect cell viability (figure 4A).

Treatment with GEA-3162 (100 μM) alone induced apoptotic cell death (annexin V binding) in MDMϕ. However, levels of apoptosis were significantly attenuated in those cells that had been subjected to previous 24 h pre-treatment with DETA/NO (10 μM) in combination with BAY 41–2272 or IBMX (*P *< 0.05 for both treatments compared to GEA-3162 treated cells; figure 4). Pre-treatment with the cell permeable cGMP analogue, 8-bromo-cGMP, also significantly attenuated subsequent GEA-3162 induced apoptosis. Similarly, DETA/NO (300 μM) afforded protection against GEA-3162 induced apoptosis and this protection was reversed in the presence of ODQ (figure 4).

## Discussion

This study reveals disparities between the ability of NO radical and NO-related species to induce apoptosis in human macrophages. Our study highlights the opposing effects of ONOO^- ^and NO is this setting and demonstrates that ONOO^-^, but not NO radical, induces apoptosis in human MDMϕ. Furthermore, our data demonstrate for the first time that the pro-apoptotic effects of ONOO^- ^are limited in human macrophages by pre-conditioning the cells with agents that act to elevate intracellular concentrations of cGMP.

During inflammation, neutrophilic leukocytes are recruited and activated during an initial wave of inflammatory cell recruitment. These inflammatory cells generate reactive oxygen and nitrogen species, which can combine to form ONOO^- ^[39], thus resulting in a pro-oxidising environment within the inflammatory site. A second wave of cellular recruitment then results in an influx of monocytes that differentiate within the inflammatory site into macrophages. These macrophages play a crucial role in phagocytosing and removing apoptotic cells during the resolution of inflammation and tissue healing [40,41]. Macrophages resident in an inflammatory site will therefore be exposed to pro-apoptotic oxidising species, including ONOO^-^, generated during the inflammatory process.

In agreement with previous studies [21], our electrochemistry and EPR studies confirm that GEA-3162 decomposes to co-generate NO and O_2_^•-^, and is therefore a ONOO^- ^generator, whilst DETA/NO liberates NO radicals in solution. In subsequent experiments, only ONOO^- ^induced apoptosis, and the level of apoptosis induced was related to the activity of the compound. ONOO^- ^is well documented to be cytotoxic in many cell types [42,43]. However, although ONOO^- ^has previously been demonstrated to be pro-apoptotic in several cell types, including murine macrophages [44,45], this is the first report of ONOO^- ^inducing apoptosis in human macrophages. Interestingly, in contrast to the actions of ONOO^-^, NO radical had no affect on cell viability. It is particularly surprising that NO radical was so ineffectual at causing either apoptosis or necrosis, even at these supraphysiological concentrations; this is in direct contrast to the observations of others who have demonstrated NO-induced apoptosis in a variety of inflammatory cells, including neutrophils [46] and murine, but not human, macrophages [9,47]. This disparity between the ability of ONOO^- ^and NO to induce apoptosis may go some way to rationalise the apparently paradoxical reports of NO being both pro- and anti-apoptotic. Our results suggest that it is NO-related species, and ONOO^- ^in particular, which might be formed in the microenvironment of the cell, that are responsible for pro-apoptotic signalling in human macrophages.

In rat thymocytes ONOO^-^-induced apoptosis was inhibited by the antioxidant, Trolox, indicating that oxidising species are the apoptotic trigger [48]. We observed that the anti-oxidant and O_2_^•- ^scavenger, SOD, actually elevated, rather than inhibited, ONOO^-^-mediated apoptosis. As our electrochemical data demonstrate, in both the absence and presence of cells, SOD effectively 'unmasks' NO production from GEA-3162 by removing O_2_^- ^from the system. Therefore, the NO liberated from GEA-3162 in the presence of SOD could itself be pro-apoptotic. However, given that we found that far higher NO concentrations released from DETA/NO failed to induce apoptosis, perhaps a more likely explanation is that hydrogen peroxide (H_2_O_2_), formed by SOD [49] during the enzymatic conversion of O_2_^•-^, is a more effective mediator of apoptosis than ONOO^- ^*per se*. Indeed, H_2_O_2 _has previously been shown to be responsible for NO-induced apoptosis in murine macrophages by activating pro-apoptotic caspase enzymes [50]. Although H_2_O_2 _can inhibit caspase enzymes under certain circumstances, this usually occurs at very high H_2_O_2 _concentrations (higher that those likely to be generated in our experiments), and only once apoptosis has already been initiated [51]. Furthermore, inhibition of caspases once apoptosis has been initiated results in a switch from apoptosis to necrosis, which did not occur in this study. It is therefore possible that H_2_O_2 _would activate, rather than inhibit, caspase enzymes in our study. If H_2_O_2 _were formed by SOD in our experimental system, it would be predicted that a combination of SOD plus catalase (to eliminate H_2_O_2_) would inhibit overall levels of apoptosis. However, although the SOD-induced elevation of apoptosis was blunted by catalase, overall levels of GEA-3162-induced apoptosis were not inhibited by a combination of SOD plus catalase. The reasons for this are not entirely clear, however, it has been reported that the enzymatic activity of catalase can be inhibited by NO via interactions with the ferric group of the enzyme [52,53]. It is therefore possible that catalase becomes inhibited by the NO unmasked from GEA-3162 in the presence of SOD and so cannot breakdown H_2_O_2_, thus apoptosis is not inhibited.

Earlier reports on the anti-apoptotic actions of NO in murine cells suggested that resistance to pro-apoptotic signals from NO, and/or NO-related species, may be a preconditioning response dependent on the anti-apoptotic properties of cGMP produced in response to the NO itself [23-25]. In common with these observations, our findings demonstrate that ONOO^-^-induced apoptosis is attenuated in human MDMϕ following prior incubation with a low concentration of NO radical in combination with BAY 41–2272 (a sGC stimulator) to augment cGMP production, or with IBMX (a PDE inhibitor) to prevent cGMP breakdown. Similarly, the cell permeable cGMP analogue, 8-bromo-cGMP, and a higher concentration of the NO donor compound, DETA/NO, also reduced apoptosis following subsequent exposure to ONOO^-^. As the cGMP-elevating agents were washed out of the cells in advance of ONOO^- ^treatment, we believe this to be a cGMP-dependent pre-conditioning mechanism that protects human MDMϕ from subsequent apoptosis. That this pre-conditioning response is dependent on cGMP signalling, rather than NO radicals *per se*, is further underlined by the observation that the sGC inhibitor, ODQ, reversed the protection against ONOO^-^-induced apoptosis afforded by higher concentrations of DETA/NO.

In rodent macrophages, the observation that cGMP primes cells against subsequent pro-apoptotic signals has been proposed as a self-defence mechanism against high iNOS-derived NO concentrations generated by macrophages during host defence against invading pathogens [24,25]. Recently, it was reported that eNOS-derived NO plays a critical role in promoting iNOS induction and function in response to lipopolysaccharide (LPS) treatment in rodent macrophages and blood vessels [54,55]. Thus, it appears that during host defence, auto-regulation between NOS isoforms allows lower concentrations of NO to induce the production of supraphysiological NO concentrations whilst simultaneously affording cGMP-dependent cellular protection against these high NO concentrations. Therefore it seems that NO is able to provide the cell with a means to finely balance pro- and anti- apoptotic NO signals. However, because NO production by human macrophages is either absent or exceedingly low [56-58], self-defence is unlikely to account for the phenomenon in human cells. The studies conducted in murine cells used relatively high concentrations (~100 μM) of NO donor compounds to induce cytoprotection and crucially, the concentration of NO radical generated by the NO donor compounds was not measured in such studies. From our electrochemistry data we have estimated that the concentration of NO released from 10 μM DETA/NO is likely to be in the nM range and arguably a more realistically physiological model of endogenous endothelial-derived NO. Our observations in human cells could, therefore, represent a cGMP-dependent mechanism by which, in the disease-free state, eNOS-derived NO from the healthy endothelium is able to limit apoptosis in macrophages. Protecting macrophages against the pro-apoptotic oxidising milieu of an inflammatory site would ensure maintenance of a sufficient population of phagocytes to effectively remove those cells that have undergone apoptosis, thus promoting the successful resolution of inflammation and preventing the tissue damage that can occur when apoptotic cells remaining *in situ *eventually rupture their membranes and release their histotoxic contents causing widespread necrosis [59].

The downstream signalling events responsible for cGMP-dependent inhibition of apoptosis in human MDMϕ remain to be elucidated. Apoptosis involves multiple pathways and signalling cascades and is controlled by the regulation of various pro- and anti-apoptotic factors within the cell. It is likely that cGMP, possibly via activation of protein kinase G (PKG; [24]) ultimately results in a shift in the pro-/anti- apoptotic balance of the cell in favour of the later.

**Figure 5:**
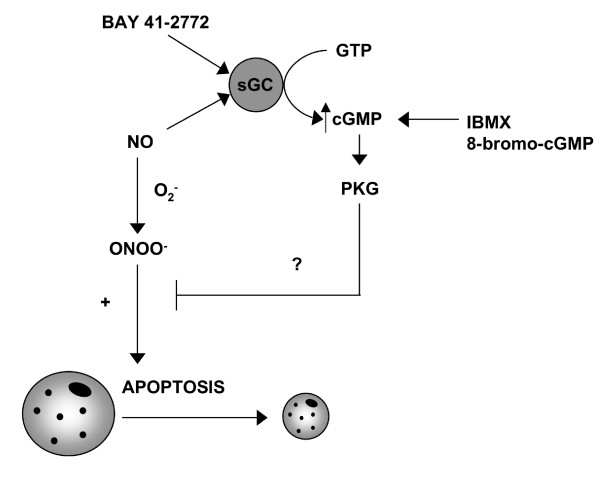
**Schematic representation of the roles of NO, ONOO^-^, & cGMP in macrophage apoptosis**. NO and O_2_^•- ^combine to form ONOO^- ^which promotes macrophage apoptosis. Agents that act to elevate cGMP are able to limit ONOO^-^-induced apoptosis.

In summary, we have demonstrated the differential ability of NO and NO-related species to induce apoptosis in human MDMϕ, and have established that ONOO^-^, but not NO radical, is pro-apoptotic in this cell type (figure 5). Furthermore, our results demonstrate the presence of a cGMP-dependent pre-conditioning mechanism to limit ONOO^-^-induced apoptosis in human macrophages.

## Abbreviations

cGMP: cyclic guanosine monophosphate; DETA/NO: (Z)-1- [2-(2-aminoethyl)-N-(2-ammonioethyl)amino]diazen-1-ium-1,2-diolate; DMSO: dimethylsulphoxide; EPR: electron paramagnetic resonance spectrometry; GEA-3162: 1,2,3,4-oxatriazolium, 5-amino-3-(3,4-dichlorophenyl)-, chloride; Hb: haemoglobin; IBMX: isobutylmethylxanthine; IMDM: Iscove's modified Dulbecco's tissue culture medium; MDMϕ: monocyte-derived macrophages; MNC: Mononuclear cells; NO: nitric oxide; O_2_^•-^: superoxide; ONOO^-^: peroxynitrite; PBS: phosphate buffered saline; PDE: phosphodiesterase; PI: propidium iodide; PRP: platelet-rich plasma; SOD: superoxide dismutase.

## Competing interests

The authors declare that they have no competing interests.

## Authors' contributions

The manuscript was written and experiments designed by CAS, AGR, and ILM. All experiments were performed by CAS and supervised by ARG and ILM who also oversaw manuscript construction together with DJW. All authors have given final approval of the version to be published.
